# IL-1β and the Intestinal Epithelial Tight Junction Barrier

**DOI:** 10.3389/fimmu.2021.767456

**Published:** 2021-10-25

**Authors:** Lauren W. Kaminsky, Rana Al-Sadi, Thomas Y. Ma

**Affiliations:** ^1^ Section of Allergy, Asthma, and Immunology, Department of Medicine, Pennsylvania State University College of Medicine, Hershey, PA, United States; ^2^ Division of Gastroenterology and Hepatology, Department of Medicine, Pennsylvania State University College of Medicine, Hershey, PA, United States

**Keywords:** interleukin-1β (IL-1β), intestinal tight junction (TJ) barrier, myosin light chain kinase (MLCK), microRNA, intestinal inflammation, NF-kappaB (NF-κB)

## Abstract

The intestinal epithelial tight junction (TJ) barrier controls the paracellular permeation of contents from the intestinal lumen into the intestinal tissue and systemic circulation. A defective intestinal TJ barrier has been implicated as an important pathogenic factor in inflammatory diseases of the gut including Crohn’s disease, ulcerative colitis, necrotizing enterocolitis, and celiac disease. Previous studies have shown that pro-inflammatory cytokines, which are produced during intestinal inflammation, including interleukin-1β (IL-1β), tumor necrosis factor-α, and interferon-γ, have important intestinal TJ barrier-modulating actions. Recent studies have shown that the IL-1β-induced increase in intestinal TJ permeability is an important contributing factor of intestinal inflammation. The IL-1β-induced increase in intestinal TJ permeability is mediated by regulatory signaling pathways and activation of nuclear transcription factor nuclear factor-κB, myosin light chain kinase gene activation, and post-transcriptional occludin gene modulation by microRNA and contributes to the intestinal inflammatory process. In this review, the regulatory role of IL-1β on intestinal TJ barrier, the intracellular mechanisms that mediate the IL-1β modulation of intestinal TJ permeability, and the potential therapeutic targeting of the TJ barrier are discussed.

## Introduction

The gastrointestinal (GI) tract is lined by a single cell layer of intestinal epithelial cells (IECs) which serve as a physical barrier against the influx of luminally located noxious substances, large hydrophilic molecules, and bacterial organisms (1, 2). The epithelial cells are interconnected to each other at the apex of the basolateral membranes by the tight junctions (TJs), which serve as both a boundary demarcating apical from basolateral membrane (referred to as the “fence” function) and as a gate or barrier to paracellular permeation of luminal contents (1, 3–5). The TJs are the apical-most intercellular junctions that prevent or regulate invasion by microorganisms, diffusion of toxins, and flux of water soluble molecules between cells, referred to as paracellular permeability (1, 2, 6–9). A complex of transmembrane proteins interact or make contact across the intercellular spaces to form TJs (1, 2, 10–12). These proteins include occludin, members of the claudin family of proteins, and the junctional adhesion molecule (JAM) family of proteins (1, 5, 12–14). Occludin plays a role in the formation and disassembly of the TJ and is involved in the regulation of the “leak pathway” or paracellular flux of large molecules (1, 15–17), whereas claudins have a greater role in controlling the “pore pathway” or paracellular flux of ions and small molecules, and JAMs are important for TJ assembly and facilitating leukocyte adhesion and migration (1, 6, 11, 18–20). The TJ proteins are also directly linked to cytoskeletal actomyosin fibers *via* cytoplasmic TJ proteins, including the zonula occludens (ZO) family (ZO-1, ZO-2, and ZO-3) (1, 6, 21–23).

A defective intestinal TJ barrier is an important contributing factor to the pathogenesis of various inflammatory conditions of the gut including celiac disease, inflammatory bowel disease (IBD), and necrotizing enterocolitis (NEC) (4–6, 24–26). Patients with Crohn’s disease have increased intestinal permeability and associated alterations in TJ proteins (6, 26). In patients with quiescent Crohn’s disease, increased intestinal permeability has been identified as an important predictor of early disease relapse (25). Therapeutic re-tightening of the intestinal TJ barrier in patients with active Crohn’s disease is associated with more rapid improvement and prolonged clinical remission, while the presence of persistently increased intestinal permeability is associated with early disease relapse (25, 27, 28), suggesting that therapeutic tightening of the intestinal TJ barrier could be an important treatment option in IBD. In addition, alterations in intestinal permeability and TJ proteins have been shown to be associated with the development of colorectal cancer (29–32).

Pro-inflammatory cytokines, including interleukin-1β (IL-1β) and tumor necrosis factor-α (TNF-α), are markedly elevated in inflammatory conditions of the gut, including IBD and NEC (33–38). Previous studies from our laboratory and others have shown that IL-1β and TNF-α at physiological concentrations cause a marked increase in intestinal epithelial TJ permeability (39–48). Recent studies suggest that, in addition to its direct immune activating effects, IL-1β also promotes intestinal inflammation by disrupting the intestinal TJ barrier and allowing increased intestinal penetration of luminal antigens (49). Consistent with such a possibility, it was recently demonstrated that the inhibition of the IL-1β-induced increase in intestinal TJ permeability prevented the dextran sodium sulfate (DSS)-induced intestinal inflammation (49). In this review, we summarize the published studies as they relate to the effect of IL-1β on the intestinal epithelial TJ barrier, the intracellular signaling pathways involved in TJ barrier modulation, the downstream molecular targets of IL-1β, and the clinical implications of IL-1β intestinal barrier modulation in the context of intestinal inflammation.

## IL-1β and Its Receptors

The IL-1 family consists of 11 distinct members which are comprised of seven immunomodulatory cytokines, IL-1α, IL-1β, IL-18, IL-36α, IL-36β, IL-36γ, IL-33, and four natural antagonists, IL-1 Receptor antagonist (IL-1Ra), IL-36Ra, IL-37, IL-38 (50). The IL-1 subfamily consists of IL-1α, IL-1β, and IL-33 (50). IL-1α and IL-1β are agonists; IL-1Ra functions as a competitive inhibitor of IL-1 (50–52). These three signaling molecules, IL-1α, IL-1β, and IL-1Ra, share 20-25% amino acid sequence homology (51). IL-1α and IL-1β are produced by immune cells such as monocytes and macrophages, but also by non-immune cells, including endothelial cells and epidermal cells, in response to different stimuli including microbes and cytokines (51). Initially produced as precursor proteins, IL-1α and IL-1β precursors undergo cleavage to generate mature proteins (51, 52). IL-1Ra can function intracellularly, such as in keratinocytes and epithelial cells, but in other cell types, such as mononuclear cells, the IL-1Ra is transported outside of the cell as secretory IL-1Ra (sIL-1Ra) where it functions to bind to and inhibit signaling through IL-1 receptors (52, 53).

There are 10 members of the IL-1 family of receptors, including IL-1 receptor type I (IL-1RI) and IL-1 receptor type II (IL-1R2) which mediate signaling by IL-1α and IL-1β with IL-1 receptor type III (IL-1R3) as a co-receptor (50, 51). IL-1R1 also binds IL-1Ra (50). IL-1R2 is released from the cell surface and binds precursor and mature forms of IL-1β, acting as a decoy receptor and inhibitor of IL-1β function (51–54), as IL-1R2 binds IL-1 but does not transduce any signal (51, 52, 54).

IL-1 and IL-1Ra levels have been shown to be elevated in intestinal inflammatory conditions such as Crohn’s disease and ulcerative colitis (33, 34). Although the levels of both IL-1 and IL-1Ra have been shown to be increased in IBD, a shift in the proportions of these proteins compared to a healthy state has been shown, with relatively less IL-1Ra compared to IL-1 (33). This shift in the IL-1Ra:IL-1 ratio correlated with disease severity (33, 53, 55), suggesting that this imbalance may play a role in the pathogenesis of IBD. A decrease in the IL-1Ra : IL-1β ratio has also been observed in canine IBD (56).

Patients with IBD have been further observed to have an abnormal increase in small intestinal permeability as measured by intestinal absorption of permeability markers such as lactulose and mannitol (57–67). Numerous clinical studies examining intestinal permeability in ulcerative colitis and Crohn’s disease have consistently shown patients with ulcerative colitis and Crohn’s disease to have significant increases in small intestinal permeability (57–67). Although patients with ulcerative colitis have intestinal inflammation that is limited to the colon, the studies have shown that these patients, similar to patients with Crohn’s disease, have significant increase in small intestinal permeability (57–67). The increase in small intestinal permeability is likely to represent in part the effect of the circulating inflammatory mediators, such as IL-1β, TNF-α, IFN-γ, and lipopolysaccharide, which cause an increase in intestinal permeability. As intestinal permeability markers are hydrophilic and do not permeate across the bilipid enterocyte plasma membrane, changes in intestinal permeability are reflective of relative “tightness” or “leakiness” of the intestinal TJ barrier or the paracellular pathways (68, 69). The abnormal increase in intestinal permeability in inflammatory diseases of the gut may be due to the intrinsic structural defect of the TJ barrier related to the underlying disease state or secondary to the effects of inflammatory process or inflammatory mediators, including IL-1β and TNF-α (7, 26, 46, 58, 68, 70, 71). IL-1β at physiologically and clinically relevant concentrations, as seen in IBD and NEC, has been shown to cause an increase in intestinal epithelial TJ permeability (33, 34, 38, 72–74). Interestingly, the inhibition of the IL-1β-induced increase in intestinal permeability in an animal model of colitis was found to be protective against the development of colitis (49), suggesting the possibility that IL-1β-induced disruption of the intestinal TJ barrier contributes to the intestinal inflammation process. It should also be noted that in the setting of ischemic injury to the small intestine, polymorphonuclear leukocytes (PMNs) were shown to enhance intestinal epithelial barrier function in an IL-1β-dependent manner (75). In this model system, it was suggested that the PMN enhancement of intestinal epithelial barrier function was mediated by IL-1β-induced upregulation of cyclooxygenase-2 (COX-2) (75). Thus in the setting of ischemic injury of intestine, IL-1β appears to mediate the PMN-protective effect. In a mouse model of colitis, IL-1β was shown to be involved in mediating tissue repair during resolution of colitis (76).

There have been very few published reports related to the role of IL-1α on the intestinal TJ barrier. In the setting of ischemic-reperfusion injury secondary to burn, IL-1α was shown to ameliorate the burn-induced increase in intestinal permeability (77). In a mouse model of colitis, IL-1α was predominantly produced by IECs and functioned as an inflammatory mediator with associated increased colonic inflammation (76). In the following sections, the intracellular and molecular mechanisms of the IL-1β modulation of the intestinal TJ barrier are reviewed.

## Mechanisms of the IL-1β-Induced Modulation of the Intestinal TJ Barrier

### Regulatory Role of Canonical and Non-Canonical Nuclear Factor-κB (NF-κB) Signaling Pathway in Intestinal TJ Barrier Regulation

In mouse models of colitis and in IBD patients, NF-κB has been shown to play an important role in mediating intestinal inflammation (78, 79). NF-κB activation has been found in macrophages and in IECs from biopsy samples from patients with IBD, and the level of activated NF-κB correlated with the severity of inflammation (78). The NF-κB activity was associated with production of the pro-inflammatory cytokines IL-1, IL-6, and TNF-α (79). IL-1 induced nuclear translocation of NF-κB mediated the activation and transcription of inflammatory genes (80, 81), with other studies showing that specifically IL-1β induced the cytoplasmic to nuclear translocation of NF-κB (39, 82–84).

NF-κB signaling for transcriptional activation can occur through the classical (canonical) or the alternative (non-canonical) pathway, with both pathways leading to the nuclear translocation and activation of NF-κB and binding of DNA by dimers of five proteins: p50, p52, p65 (RelA), RelB, and c-Rel (85–87). In the canonical pathway, degradation of the inhibitory IκB protein occurs following phosphorylation of the IκB kinase (IKK) complex that consists of the catalytic kinases IKKα and IKKβ and the regulatory subunit, IKKγ (NEMO) (86, 87). IKKβ phosphorylates IκB at specific serine residues to mediate degradation of this inhibitory protein (86, 87). Phosphorylated IκB is ubiquitinated and degraded by the proteasome, which allows NF-κB p50/p65 heterodimers to translocate from the cytoplasm to the nucleus to activate the inflammatory genes (86, 87).

**Figure 1:**
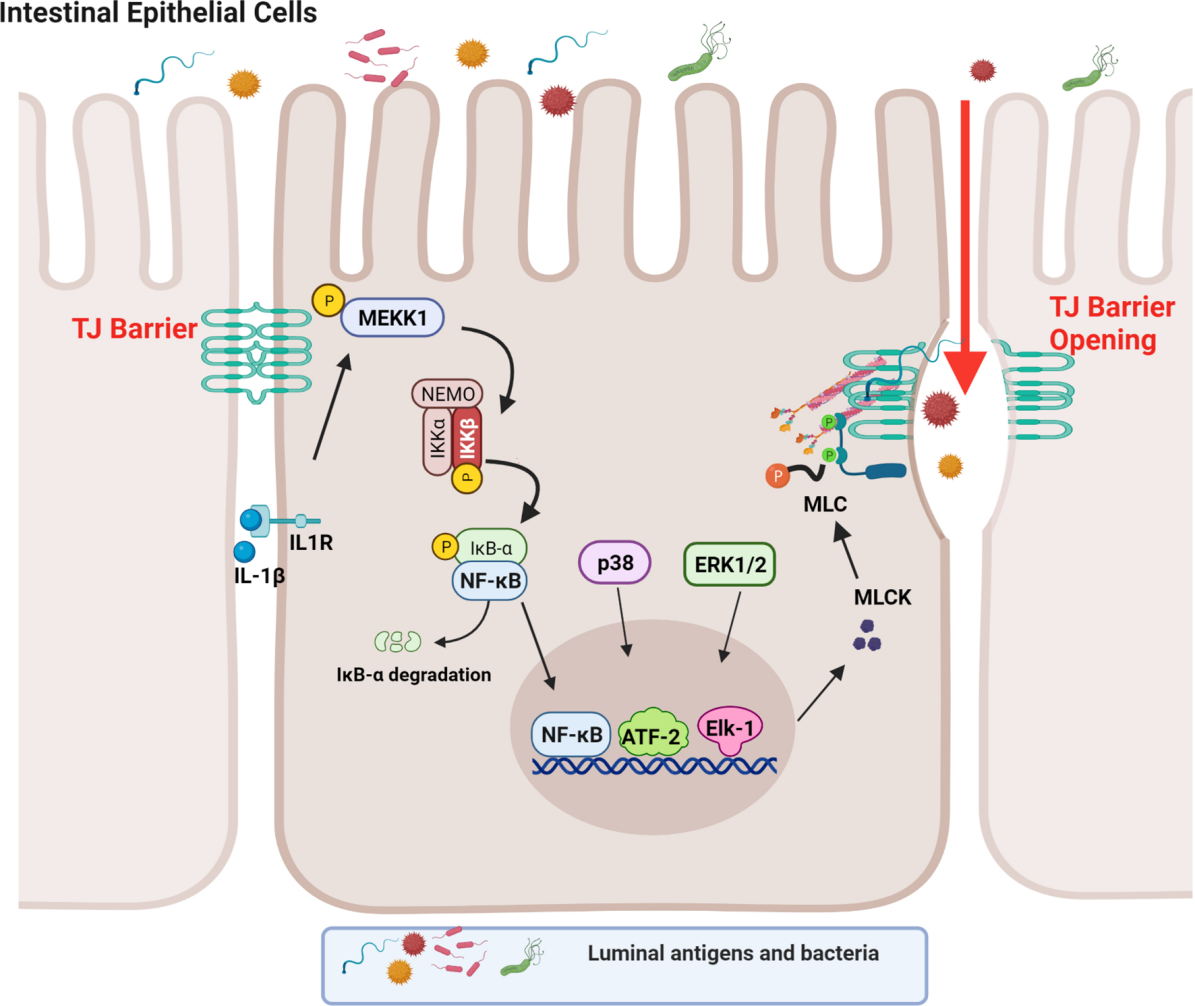
Schematic diagram depicting the involvement of MEKK-1, IKK-β, p38 kinase, ERK1/2, and NF-κB in the interleukin-1β (IL-1β)-induced increase in myosin light chain kinase (MLCK) gene activity in intestinal epithelial tight junction (TJ) permeability. (Created with BioRender.com).

Although both the canonical and non-canonical NF-κB pathways were found to be activated in IECs by IL-1β in the context of TJ barrier regulation, only the canonical pathway (NF-κB p50/p65) was involved in the IL-1β-induced dysfunction of the TJ barrier (88). Supporting the requirement for the canonical NF-κB pathway, inhibition of NF-κB p50/p65 activation, but not p52 or p100, prevented the IL-1β-induced increase in intestinal TJ permeability (39). In regards to the canonical NF-κB pathway, IL-1β caused a rapid degradation of IκBα leading to the cytoplasmic-to-nuclear translocation of NF-κB p50/p65 and activation of the target gene, myosin light chain kinase (MLCK) gene (39, 88–90). IL-1β rapidly produced a time-dependent activation of the upstream regulatory protein kinase, mitogen-activated protein kinase kinase kinase-1 (MEKK-1), and NF-κB-inducing kinase (NIK) (88, 91). Although IL-1β caused activation of both MEKK-1 and NIK, the canonical NF-κB pathway (NF-κB p50/p65) activation was mediated by only MEKK-1 and not NIK activation (88). The siRNA induced knockdown of MEKK-1, but not of NIK, inhibited the IL-1β-induced activation of NF-κB p50/p65 (88). Knockdown of p65 also inhibited the IL-1β-induced dysfunction of the TJ barrier (88), confirming the requirement for the canonical NF-κB pathway. IL-1β also caused an activation and increase in nuclear translocation of the p52 subunit (non-canonical NF-κB pathway) with binding of p52 to the κB DNA binding site; however, the p52 subunit was not required for the IL-1β-induced dysfunction of the TJ barrier (88). These findings confirmed that the IL-1β-induced increase in intestinal TJ permeability was mediated by the MEKK-1-dependent activation of NF-κB p50/p65 (**Figure 1**).

As an integral component of IL-1β regulation of the canonical NF-κB pathway, IL-1β caused a rapid activation of IKK catalytic subunits, IKKα and IKKβ, in Caco-2 intestinal epithelial monolayers and in mouse IECs (88, 92–94). The IL-1β-induced activation of IEC IKKα and IKKβ was dependent on MEKK-1 activation (88). As for the specific IKK subunit involved in the NF-κB p50/p65 activation and the increase in TJ permeability, IKKβ was the dominant subunit that was required for the NF-κB activation and the subsequent increase in TJ permeability in the intestinal monolayers (88). Interestingly, IKKα also played a role in the increase in TJ permeability and the targeted knockdown of IKKα resulted in partial inhibition of NF-κB activation and an increase in TJ permeability (88). Thus, the IL-1β-induced increase in intestinal TJ permeability was mediated by the MEKK-1-induced activation of IKK catalytic subunits IKKβ and IKKα, and IKKβ-induced phosphorylation and degradation of enterocyte inhibitory κB protein and activation of NF-κB p50/p65 (**Figure 1**) (88).

TNF-α is an additional pro-inflammatory cytokine that has been implicated in IBD and causes increased intestinal epithelial TJ permeability (40, 95, 96). The TNF-α-induced increase in intestinal TJ permeability was also dependent on NF-κB p50/p65 activation (40, 97–103). However, important differences are noted concerning how the enterocyte NF-κB p50/p65 was activated and how the TJ barrier was regulated. The TNF-α-induced activation of NF-κB was mediated by the NIK-induced activation of the IKKα homodimer and not IKKβ catalytic subunit (104); as described above, the IL-1β-induced increase in TJ permeability was regulated by MEKK-1 activation of IKKβ catalytic subunit (88).

### Involvement of MLCK in IL-1β Intestinal TJ Barrier Modulation

It is well-established that MLCK protein level and activity are markedly increased in intestinal tissues obtained from patients with IBD (105). The increase in MLCK protein level was found to correlate with the severity of intestinal inflammation (105). MLCK is a Ca^2+^-calmodulin activated serine/threonine kinase that regulates actomyosin rearrangement and cell contraction in various cell types including smooth, cardiac, and skeletal muscle and in non-muscle cells (106–111). Two long non-muscle isoforms, MLCK1 (full-length long MLCK) and MLCK2 (which lacks a single exon), are predominantly expressed in intestinal epithelial cells and play a central role in modulating various cell functions (112–114). MLCK1 is also known to regulate the intestinal TJ barrier function *via* phosphorylation of myosin II regulatory light chain (MLC) at threonine-18 and/or serine-19 leading to peri-junctional actomyosin ring contraction, mechanical retraction of apical membrane and pulling apart of the TJ complex, and opening of the intestinal TJ barrier (109, 115–117). Previous studies have shown that the IL-1β-induced increase in intestinal epithelial TJ permeability in Caco-2 intestinal epithelial monolayers and mouse small intestine *in vivo* was mediated by an increase in MLCK gene activity and protein expression and an increase in MLCK enzymatic activity (84) (45, 46, 118–120). In these studies, IL-1β caused a rapid activation of MLCK gene activity and increase in MLCK protein expression; inhibition of MLCK activity by pharmacologic inhibitors or by siRNA-induced knockdown prevented the IL-1β-induced increase in intestinal epithelial TJ permeability (84, 121). The IL-1β-induced increase in MLCK gene activity and protein synthesis was mediated by the activation of NF-κB p50/p65, involving the MEKK-1/IKKβ/NF-κB p65 axis as described above (**Figure 1**) (84). In these studies, targeted knockdown of MEKK-1, IKKβ, or NF-κB p65 prevented the IL-1β-induced increase in MLCK gene activity and increase in intestinal epithelial TJ permeability, confirming the regulatory role of MEKK-1, IKKβ and the NF-κB p50/p65 axis on MLCK gene activation (88). Zhai and colleagues recently showed that low-fat yogurt prevented the IL-1β-induced intestinal barrier disruption by inhibiting the upregulation of MLCK gene expression in Caco-2 cells (122). These studies also showed that the IL-1β-induced increase in NF-κB activation is inhibited by low-fat yogurt application to Caco-2 cells (122). Collectively, these studies show that IL-1β-induced increases in MLCK gene activity and protein synthesis are required for increased intestinal TJ permeability. The NF-κB p50/p65 signaling pathway regulates the TJ barrier by targeting MLCK gene activation (**Figure 1**).

### Role of Mitogen-Activated Protein Kinases (MAPKs) Signal Transduction Pathways and Other Transcription Factors in the IL-1β-Induced Increase in Intestinal TJ Permeability

MAPKs are serine-threonine protein kinases that mediate cellular activities including proliferation, differentiation, apoptosis, survival, inflammation, and innate immune responses (123, 124). There are at least three groups of MAPKs identified including extracellular signal-regulated kinases (ERK), the p38 MAPKs, and the cJun NH_2_-terminal kinases (JNK) (125). It is well-established that IL-1β and other pro-inflammatory cytokines can activate MAPK signal transduction pathways. Previous studies have shown that the p38 kinase signaling cascade also contributes to IL-1β-induced increased MLCK expression and increased intestinal TJ permeability *in vitro* and *in vivo* (46). These studies found that IL-1β induced a rapid phosphorylation and activation of p38 kinase and downstream activation of a p38-dependent transcription factor, activating transcription factor-2 (ATF-2); the activated ATF-2 translocated to the nucleus and attached to the binding motif on the MLCK promoter region, leading to MLCK gene activation and protein synthesis, and MLCK-dependent opening of the intestinal TJ barrier (46). The targeted knockdown of p38 kinase and ATF-2 inhibited the IL-1β-induced activation of MLCK gene and increased intestinal TJ permeability in Caco-2 monolayers and in mouse small intestine (46). Thus, in addition to NF-κB, ATF-2 also played an important role in the regulation of the IL-1β-induced activation of MLCK gene and increase in MLCK protein synthesis. Other studies have shown that p38 kinase activation is required for IL-1β-induced regulation of the TJ protein claudin-2 in a rat hepatic injury model (126, 127).

In addition to NF-κB and ATF-2, transcription factor Elk-1 has also been shown to contribute to IL-1β-induced increase in intestinal TJ permeability (46, 128). IL-1β has also been found to cause a rapid activation of Elk-1 in intestinal epithelial cells (128, 129). Upon activation, Elk-1 translocated to the nucleus and attached to the cis-binding motif on the MLCK promoter region, leading to the activation of MLCK gene and an increase in TJ permeability (128). In aggregate, these studies suggest that IL-1β induces the activation and binding of transcription factors NF-κB p50/p65, ATF-2, and Elk-1 to the MLCK minimal promoter region in close proximity to each other, and in concert, activate the MLCK gene and produce an opening of the TJ barrier (**Figure 1**) (128).

### Role of TJ Proteins and microRNAs (miRNAs) and Therapeutic Targeting of the Intestinal TJ Barrier

Intestinal epithelial TJ barrier function is maintained by intercellular TJs, including multi-protein complexes that seal the space between adjacent cells at the boundary of the apical and lateral membrane surfaces of adjacent epithelial cells (1, 130–133). TJs, which are the apical-most intercellular junctions, regulate the paracellular flux of ions and solutes, referred to as the “gate function” (8, 130–133). Related to the gate function, disturbances in TJ barrier function result in increased TJ or paracellular permeability and increased exposure of underlying intestinal tissue and other organ systems to noxious luminal antigens.

The TJ complex consists of integral transmembrane proteins such as occludin, claudins, and JAMs, as well as cytosolic scaffold proteins, such as ZO-1, ZO-2, and ZO-3, which in turn anchor the transmembrane proteins to the actin cytoskeleton (5, 11, 134). Previous studies from our laboratory and others have shown that occludin plays an important regulatory role in restricting the paracellular flux of large macromolecules *via* a non-restrictive or “leak” pathway and that occludin depletion leads to a preferential increase in paracellular flux of macromolecules (15, 131–133, 135, 136). In addition to targeting MLCK gene activation, IL-1β-induced increase in intestinal TJ permeability has also been found to be dependent on post-transcriptional degradation of occludin mRNA (49).

**Figure 2:**
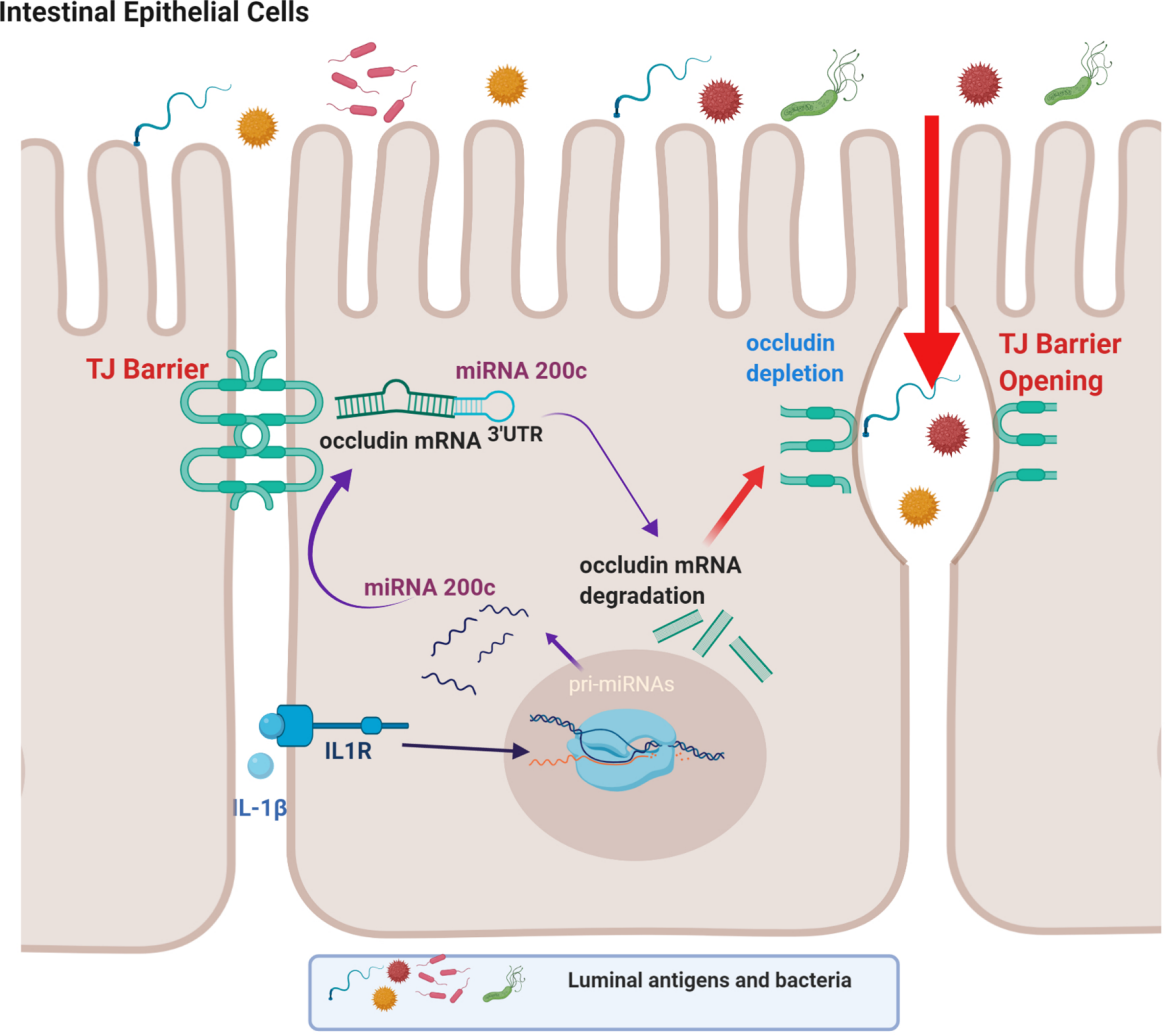
Schematic diagram showing the involvement of the microRNA-200c-3p-induced degradation of occludin mRNA in interleukin-1β (IL-1β)-induced increase in intestinal epithelial tight junction permeability. (Created with BioRender.com).

IL-1β treatment has been shown to selectively decrease the occludin level in Caco-2 monolayers and mouse intestinal epithelial cells in live mice without affecting other transmembrane TJ proteins (49). In these studies involving Caco-2 monolayers and live mice, IL-1β caused a rapid increase in miRNAs which bind to occludin mRNA (49). MiRNAs are small, single-stranded non-coding RNAs that bind to miRNA response elements on the 3’ untranslated region (3’-UTR) of mRNA to induce mRNA degradation or suppression of translation (49, 137, 138). It was found that IL-1β caused a rapid and marked increase in miR-200c-3p in IECs (49). Bioinformatics analysis predicted high probability of miR-200c-3p binding to the occludin 3’-UTR and post-transcriptional regulation. These studies also showed that increasing miR-200c-3p expression in IECs was sufficient to cause an increase in intestinal TJ permeability in Caco-2 monolayers and mice and that treatment with antagomirs, which inhibit or silence miR-200c-3p, inhibited the IL-1β-induced decrease in occludin expression and increase in intestinal epithelial TJ permeability (49). This work provides support for the cause-and-effect relationship between post-transcriptional miR-200c-3p degradation of occludin mRNA and increase in TJ permeability (**Figure 2**) (49).

**Figure 3:**
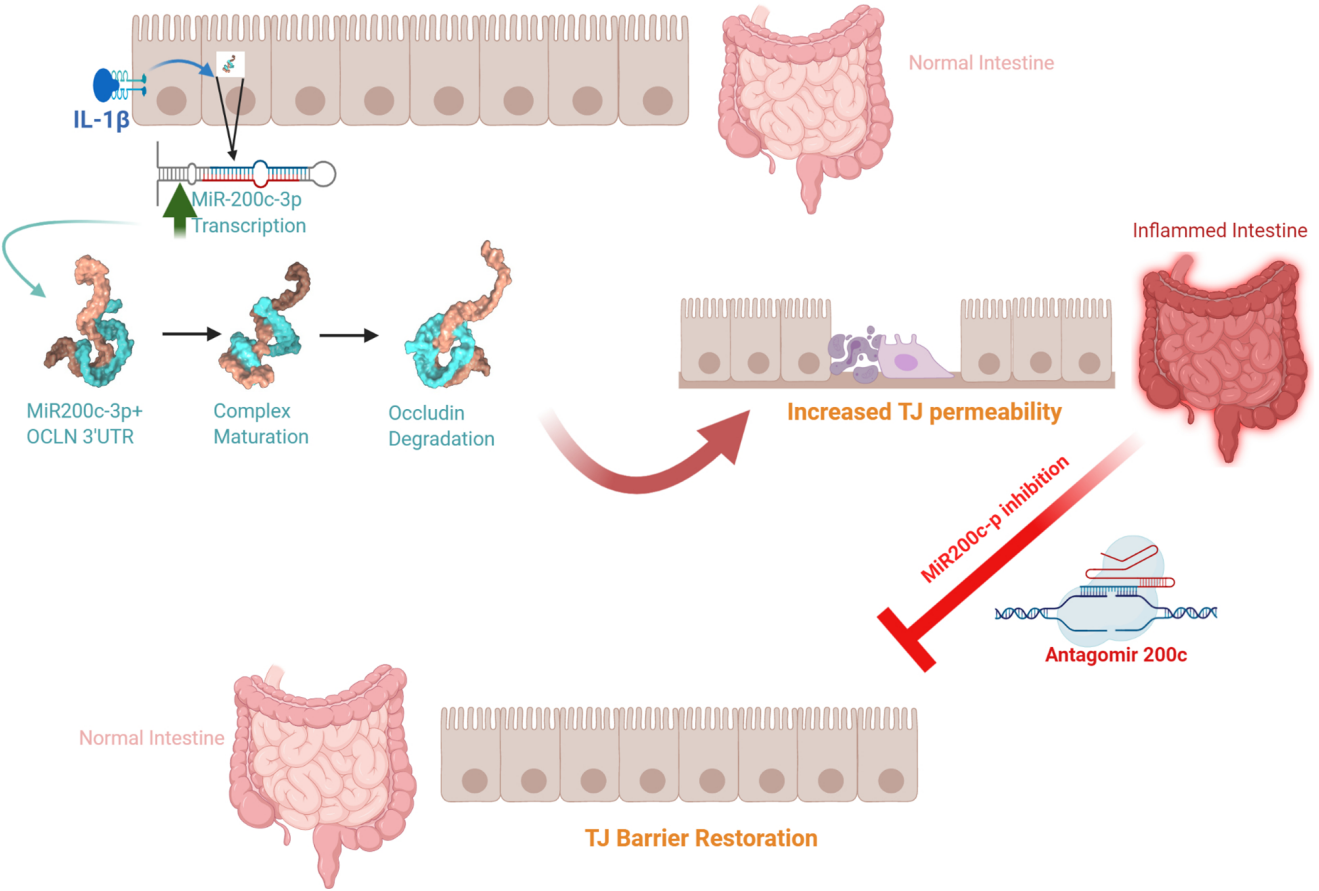
Schematic diagram of the potential role of the interleukin-1β (IL-1β)-induced increase in microRNA-200c-3p expression and intestinal tight junction (TJ) permeability in modulating intestinal inflammation. (Created with BioRender.com).

The defective intestinal TJ barrier is an important pathogenic factor contributing to intestinal inflammatory processes in IBD, NEC, and celiac disease (57, 139–141). Previous work has also investigated the potential role of elevated IL-1β present in intestinal inflammation and its contribution to TJ barrier disturbance or “leaky gut” through targeting of miR-200c-3p expression (49). IL-1β expression was shown to be markedly elevated in colonic tissues of ulcerative colitis patients and directly correlated with an increase in miR-200c-3p expression (49). Interestingly, the organoids generated from colonic tissue from ulcerative colitis patients also showed marked increase in IL-1β and miR-200c-3p expression compared to the organoids from the healthy control patients (49). These studies indicated that IL-1β and miR-200c-3p expression was markedly elevated in colonic tissue and epithelial cells from patients with ulcerative colitis and also suggested the possibility that miR-200c-3p may be targeted to inhibit the IL-1β-associated increase in intestinal TJ permeability and treat intestinal inflammation. This possibility was tested in proof-of-concept studies using DSS-induced colitis as a murine model of colitis (49). DSS oral administration caused a marked increase in intestinal tissue IL-1β and miR-200c-3p expression and was further associated with a decrease in occludin expression and increase in colonic permeability. The oral administration of antogomiR-200c inhibited DSS-induced increase in intestinal tissue miR-200c-3p levels, decrease in occludin levels, increase in colonic permeability, and development of colitis (49). Taken together, these studies demonstrate that therapeutic targeting of the IL-1β-induced increase in miR-200c-3p expression and intestinal permeability is effective in preventing DSS-induced intestinal inflammation (**Figure 3**). Additional studies have examined the impact of IL-1β on intestinal occludin expression using a dog model of IBD. Ogawa et al. reported an increase in the IL-1β:IL-1Ra ratio in the colonic mucosa of dogs with IBD; this increase was correlated with a decrease in occludin mRNA expression, intestinal barrier dysfunction, and colonic inflammation in a dog model of IBD (73).

Although dysregulation of different types of claudin proteins has been linked to the pathogenesis of IBD, the observed effect of IL-1β on claudin proteins has been shown to be limited and inconsistent (30, 142–148). Claudins are a transmembrane protein family comprised of at least 27 members (149). In the GI tract, only a few of the claudins are expressed and known to regulate intestinal TJ barrier function, including claudin-1, claudin-2, claudin-3, claudin-4, claudin-5, claudin-8, claudin-12, and claudin-18 (150–153). A previous study showed that IL-1β did not affect the expression or the distribution of claudin-1, claudin-2, claudin-3, claudin-4, or claudin-5 (39). In contrast, Maria-Ferreira et al. showed that IL-1β induced an increase in Caco-2 TJ permeability that was associated with a decrease in claudin-1 expression and disruption in occludin junctional localization (154). Haines et al. reported that the IL-1β-induced increase in intestinal TJ permeability was mediated in part by claudin-3 downregulation and translocation from the junctional localization into the nucleus; however, expression levels of ZO-1, occludin, claudin-1, claudin-4, and claudin-15 remained unchanged (121). This study also found that IL-1β induced an association of β-catenin with the claudin-3 promoter in an MLCK-dependent manner (121). Consistent with these findings, other reports have shown that Wnt signaling (and subsequent β-catenin nuclear accumulation) induced claudin-3 downregulation, and subsequently led to an increase in intestinal permeability (155). Although Guo and colleagues found that there was no change in ZO-1 protein level in response to IL-1β, they found an increase in claudin-1 expression and a decrease in occludin expression (82). Wang et al. showed that ZO-1, claudin-1, and claudin-7 junctional localization was also disrupted by IL-1β treatment in Caco-2 cells, and was associated with an increase in intestinal TJ permeability (156). The effect of IL-1β-induced increase in Caco-2 TJ permeability was also proposed to be mediated by an increase in p38 kinase phosphorylation, leading to an increase in MLCK gene and protein expression and indirectly influencing the junctional localization of ZO-1, claudin-1, and claudin-7 (156). The results of studies evaluating the effect of IL-1β on claudin proteins have been inconsistent; some studies suggested that IL-1β has no effect, while others have suggested that IL-1β downregulates claudin-3 or causes an alteration in ZO-1, claudin-1, and claudin-7 junctional localization in a MLCK-dependent manner.

## Conclusion and Future Perspectives

IL-1β is a pluripotent pro-inflammatory cytokine that is elevated in various inflammatory diseases, including NEC, irritable bowel syndrome, infectious diarrhea, and IBD. Accumulating evidence has shown that IL-1β plays an important pathogenic role in the intestinal inflammation process in these diseases (51, 157, 158). A key hallmark of IBD is intestinal epithelial TJ barrier disruption manifested by an increase in intestinal permeability (71, 140, 159); however, the precise factors which contribute to the defective intestinal TJ barrier and the role IL-1β plays in the barrier defect remains unclear. In this review, we highlight recent scientific advances that demonstrate the disruptive influence of IL-1β on the intestinal TJ barrier. We further provide insight into the intracellular signaling process, the effector and the TJ proteins involved, and the potential for therapeutic targeting of IL-1β-induced disruption of the intestinal TJ barrier to prevent or treat intestinal inflammation. In summary, IL-1β is markedly elevated in intestinal inflammation associated with ulcerative colitis and other inflammatory diseases, IL-1β causes a rapid and marked increase in intestinal TJ permeability, IL-1β-induced increase in TJ permeability is mediated in part by the activation of the canonical NF-κB pathway, MLCK gene activation and post-transcriptional degradation of occludin mRNA, and therapeutic targeting of the IL-1β-induced increase in intestinal TJ permeability is effective in preventing colitis in a murine model of colitis. Thus, IL-1β modulation of the intestinal TJ barrier represents an important potential pathogenic mechanism contributing to the observed increase in intestinal permeability in various inflammatory conditions of the gut and is a potential therapeutic target to prevent or treat intestinal inflammation.

## Author Contributions

LWK wrote and edited the manuscript. RA-S edited and revised the manuscript and created the figures. TYM conceived, edited and revised the manuscript and approved final version of this manuscript. All authors contributed to the article and approved the submitted version.

## Funding

This research project was supported by the National Institute of Diabetes and Digestive and Kidney Diseases grants R0-1-DK-64165-01 (TYM), R0-1-DK-106072-01 (TYM), R01-DK-121073-01 (TYM), R01-DK-081429 (TYM), the J. Lloyd Huck Chair in Medicine endowment from the Penn State College of Medicine (TYM), and the Department of Medicine Inspiration Award from the Penn State College of Medicine (RA-S).

## Conflict of Interest

The authors declare that the research was conducted in the absence of any commercial or financial relationships that could be construed as a potential conflict of interest.

## Publisher’s Note

All claims expressed in this article are solely those of the authors and do not necessarily represent those of their affiliated organizations, or those of the publisher, the editors and the reviewers. Any product that may be evaluated in this article, or claim that may be made by its manufacturer, is not guaranteed or endorsed by the publisher.
